# Evaluation of shaping ability of different glide path instruments: a micro-computed tomography study

**DOI:** 10.1186/s12903-023-03529-3

**Published:** 2023-10-24

**Authors:** Merve Yeniçeri Özata, Seda Falakaloğlu, Ali Keleş, Özkan Adıgüzel, Mustafa Gündoğar

**Affiliations:** 1 Department of Endodontics, School of Dentistry, Dicle University, Diyarbakır, Turkey; 2 Department of Endodontics, School of Dentistry, İstanbul Health and Technology University, İstanbul, Turkey; 3 Department of Endodontics, School of Dentistry, Bolu Abant İzzet Baysal University, Bolu, Turkey; 4 Department of Endodontics, School of Dentistry, Ondokuz Mayıs University, Samsun, Turkey; 5 Department of Endodontics, School of Dentistry, İstanbul Medipol University, İstanbul, Turkey

**Keywords:** Glide path, Shaping ability, TruNatomy glider, WaveOne Gold glider, ProGlider

## Abstract

**Background:**

This study aimed to compare the shaping ability of different instruments, TruNatomy Glider (TRN-G), WaveOne Gold Glider (WOG-G), and ProGlider (Pro-G) using micro-computed tomography (micro-CT).

**Methods:**

The mesial canals of 27 mandibular molars with two separate mesial canals and moderate curvature were included in this study [n = 27 mesiobuccal (MB) and mesiolingual (ML) root canal]. According to the manufacturer’s instructions, the glide path was created with TRN-G, WOG-G, and Pro-G glide path instruments (n = 9 MB and ML root canal in each group). Micro-CT scanning was performed before and after preparation. Mesiodistal (MD) and buccolingual (BL) transportation and the centering ratio were measured at three levels within the canal (3, 5 and 7 mm). A three-way robust ANOVA was used to compare the parameters.

**Results:**

TRN-G showed significantly greater transportation in the MD direction than the other instruments throughout the root canal (overall root canal) (p < 0.05). The best centering ability in the BL direction was shown by the WOG-G, regardless of level within the canal and canal distinction (MB vs. ML) (p < 0.05). There was no significant difference between groups according to the level within the canal and canal parameters (p > 0.05). Whether the root canal was MB or ML did not affect centering or transportation (p > 0.05).

**Conclusions:**

Glide path instruments can be used to shape moderately curved canals with minimal apical transportation and better centering ability. All three tested glide path files can used safely before the shaping file.

## Introduction

The creation of a glide path in endodontics, defined as ‘’the secret to rotary safety’’ in 2006 [1], has become an indispensable step in root canal preparation with nickel-titanium (NiTi) systems in recent years. The use of glide path instruments must be compatible with the original root canal anatomy, allowing the use of file systems with larger sizes and taper in subsequent steps [2]. Glide path preparation can be done with a stainless steel hand file or with low-taper NiTi instruments [3]. NiTi glide path instruments save time and simplify glide path creation, especially in curved root canals [4, 5] and reduce working time, providing more comfort for clinicians and patients.

Glide path instruments that are currently available are made from various NiTi metal alloys that use continuous rotary or reciprocating motions. The following glide path instruments were used in this study: WaveOne Gold Glider (WOG-G; Dentsply Sirona, Switzerland), ProGlider (Pro-G; Dentsply Sirona), and TruNatomy Glider (TRN-G; Dentsply Sirona;). WOG-G is a reciprocating file system made of a thermo-mechanically treated alloy termed “Gold” by the manufacturer. This instrument has a 0.15-mm tip diameter and variable 2–6% tapers with maximum flute diameters at D1 of 0.170 mm, D8 0.413, and D16 0.850 mm and a parallelogram-shaped cross-section with two cutting edges [6]. Pro-G is used with a continuous rotary motion and is manufactured from heat-treated NiTi M-Wire alloy [7]. This instrument has a 0.16 mm tip diameter and progressive taper (0.02 at tip level up to 0.085), aiming to enlarge the coronal and middle portions of the root canal, and a square cross-sectional shape [7, 8]. The TRN rotary system is a set of instruments with a maximum fluted diameter of 0.8 mm NiTi and proprietary heat treatment. TRN system has been claimed to provide slim shaping instrumentation because of its geometry, regressive tapers, and slim design. TRN-G has an off-center parallelogram cross-section design and variable taper (size 17, 0.02v taper) [9].

A systematic review reported that glide path preparation steps reduce canal transportation during root canal preparation. In particular, NiTi mechanized glide path instruments preserve the original canal anatomy better than hand glide path preparation [10]. Centering and transportation generated by glide path instruments may increase during subsequent shaping [11]. The best way to evaluate the changes in canal anatomy that differences between mechanized glide path instruments may cause after preparation is via micro-computed tomographic (micro-CT) scanning [12, 13]. However, few studies have compared the shaping ability of different glide path instruments without shaping files [11, 14–16].

The present in vitro study aimed to evaluate the shaping ability of different glide path instruments in preparing mesial root canals of extracted mandibular molars using micro-CT imaging. The null hypothesis was that there are no differences in preserving original root canal anatomy when using WOG-G, Pro-G, and TRN-G glide path instruments.

## Materials and methods

### Sample size calculation

Based on a previous study, we planned to study a total of 21 root canals, 7 in each group, with 80% confidence (1-α), 80.8% test power (1-β), and f = 0.745 effect size [11]. Two more root canals per group were added to the study given the dropout rate (18%) due to cracks and fractures that may occur in the tooth after root canal preparation. At the end of the study, when seven root canals had been completed in each group, the post-hoc power was 89.4% with 95% confidence (1-α) and f = 0.4 effect size (G*Power 3.1, Heinrich Heine, Düsseldorf, Germany).

### Sample selection

One hundred and sixty-five human mandibular molar teeth, each with a closed apex, no cracks, and no caries, extracted for periodontal reasons, were selected from a pool of teeth. Then, all tooth roots were wrapped with teflon tape. Then polyvinyl siloxane impression material (heavy body) (Zetaplus, Zhermack SpA, Italy) was prepared and formed into arcs, and 20 teeth were placed in each arc. The CBCT images were obtained with I-CAT Vision TM (Imaging Science International, Hatfield, USA). Imaging parameters were set to 120 kV, 5 mA, 8.9 s, and a field of view measuring 16 × 13 cm at 0.3 voxels. The images were transferred a software, and the curvature of teeth was measured (Image J, 1.36b; National Institutes of Health, Bethesda, MD) according to the method of Schneider [17]. According to this method, in MD and BL view, a first line was drawn parallel to the long axis of the canal in the coronal third and a second line was drawn from the apical foramen. The acute angle formed at the intersection of these lines showed the curvature angle of the root. Like this, teeth with curved mesial roots of 10 to 20 degrees (moderate curvature) were identified, and 62 of these were selected for root preparation, each tooth having two separate canals in the pulp chamber that ended as two separate canals (Vertucci type 4) [18]. After the curvature evaluation, the curvature radius of all teeth that met the criteria were measured. To determine the curvature radius, a circle tangent to both lines used in the measurement of the angle of curvature was drawn. The radius of this circle gave the curvature radius. An endodontic access cavity was prepared for all teeth. Then, a 10-K file was advanced in the mesiobuccal and mesiolingual canals under the stereomicroscope at ×40 magnification. Teeth with an apical diameter greater than 0.10 mm were excluded. Twenty-seven teeth met the inclusion criteria. Teeth were removed from the polyvinyl siloxane model, and teflon tapes were removed.

### Sample preparation

Teeth were flattened from their crowns to create a standard measuring point at which the silicon stoppers would be placed. The tooth lengths were 17.8 ± 0.7 mm on average. The working length (WL) was determined as 1 mm behind the file seen through the apical foramen. All teeth were positioned with light body impression material (Zetaplus, Zhermack SpA, Italy) on a custom-made acrylic mould to stabilize and mimic the periodontal ligament [16]. Then each mould was numbered and randomly placed in one of three groups according to the glide path instrument to be used (n = 9 MB and ML root canal in each group). It was determined that the curvature degrees and radius measurements (both in the MD and BL view) of the samples, which were randomly divided into three groups, showed a homogeneous distribution between the groups (Table 1).

**Table 1:** Pre-instrumentation curvature and radius mean and standard deviation values of the teeth included in the groups

View	Canal	File	Curvature (°)	Radius (mm)
**MD**	**MB**	**Pro-G**	16.4 ± 3.0	6.5 ± 1.3
		**WOG-G**	15.2 ± 2.8	6.4 ± 1.1
		**TRN-G**	14.3 ± 3.0	6.4 ± 1.3
		**p**	0.308	0.970
	**ML**	**Pro-G**	16.6 ± 2.2	6.0 ± 0.9
		**WOG-G**	16.2 ± 2.3	6.0 ± 1.1
		**TRN-G**	14.7 ± 3.1	5.7 ± 0.8
		**p**	0.278	0.685
**BL**	**MB**	**Pro-G**	15.2 ± 2.8	6.0 ± 1.2
		**WOG-G**	16.5 ± 2.6	6,1 ± 1.0
		**TRN-G**	16.2 ± 3.0	5.9 ± 1.1
		**p**	0.618	0.945
	**ML**	**Pro-G**	15.4 ± 3.7	5.5 ± 0.9
		**WOG-G**	16.1 ± 2.3	6.1 ± 1.2
		**TRN-G**	14.8 ± 3.8	5.2 ± 0.7
		**p**	0.729	0.111

* ANOVA test

#### Root canal preparation

A 10-K-file was used to scout out the root canal up to the WL for all groups. A single operator performed root canal preparation with the glide path instrument following the manufacturer’s instructions. An X.Smart Plus (Dentsply Sirona, Ballaigues, Switzerland) endodontic motor was used for rotation and reciprocation movements. Each canal was irrigated with 10 ml of 5.25% sodium hypochlorite (NaOCl) (Promida, Eskişehir, Turkey) and 5 ml distilled water using a 30-gauge TruNatomy irrigation needle (Dentsply Sirona, Ballaigues, Switzerland). The features of glide path instruments included in this study was listed in Table 2.

**Table 2:** Characteristics of the glide path instruments included in this study

Instrument	Manufacturer	Alloy	Diameter/Taper	Cross-section	Kinematic
**Pro-G**	Dentsply Sirona, Switzerland	M-wire	16; variable taper (2%)	Square	Continuous rotation
**WOG-G**		Gold-wire	15; variable taper (2%)	Parallelogram	Reciprocating motion
**TRN-G**		A new thermal treatment [19]	17; variable taper (2%)	Parallelogram	Continuous rotation

Abbreviations: Pro-G, Proglider; WOG-G, WaveOne Gold Glider; TRN-G, TruNatomy Glider.

### TRN-G group

The TRN-G instrument (17./02) was set at 500 rpm and 1.5 Ncm torque. The instrument was used with gentle strokes until the working length was reached.

### WOG-G group

The WOGG (15./02–06) was used in the WaveOne mode. An in-and-out pecking motion was applied until the working length was reached. Gentle apical pressure was applied to the file according to the manufacturer’s instructions.

### Pro-G group

The Pro-G (16./02–08) instrument was used at a speed of 300 rpm and 3 Ncm torque with a continuous rotation motion. The instrument was used with gentle strokes until the working length was reached.

A glide path file was used for the MB and ML canals in each group (one file for two canals).

#### Micro-CT scanning

The teeth were scanned before and after root canal preparation with a Skyscan 1172 v.1.1.17 (Bruker micro-CT, Kontich, Belgium) device. A 0.600-degree rotation step, a 0.5 mm Al filter, 90 kV, and 105 mA were used and images with a pixel of 16.202 μm were obtained. Acquired TIFF images were reconstructed in NRecon v.1.7.1.1 (Bruker micro-CT) with 25% beam hardening correction, and three smoothing and 10 ring artifact corrections. After the reconstruction, approximately 1000 two-dimensional (2D) axial section images of each sample and canal space were obtained. The images before and after root canal preparation were superpositioned using the 3D recording function of the Data Viewer v.1.5.6.2 (Bruker micro-CT). The images were transferred to the CTAn v.1.17.7.2 (Bruker micro-CT) for 2D measurement.

#### Transportation and centering ability assessment

**Fig. 1:**
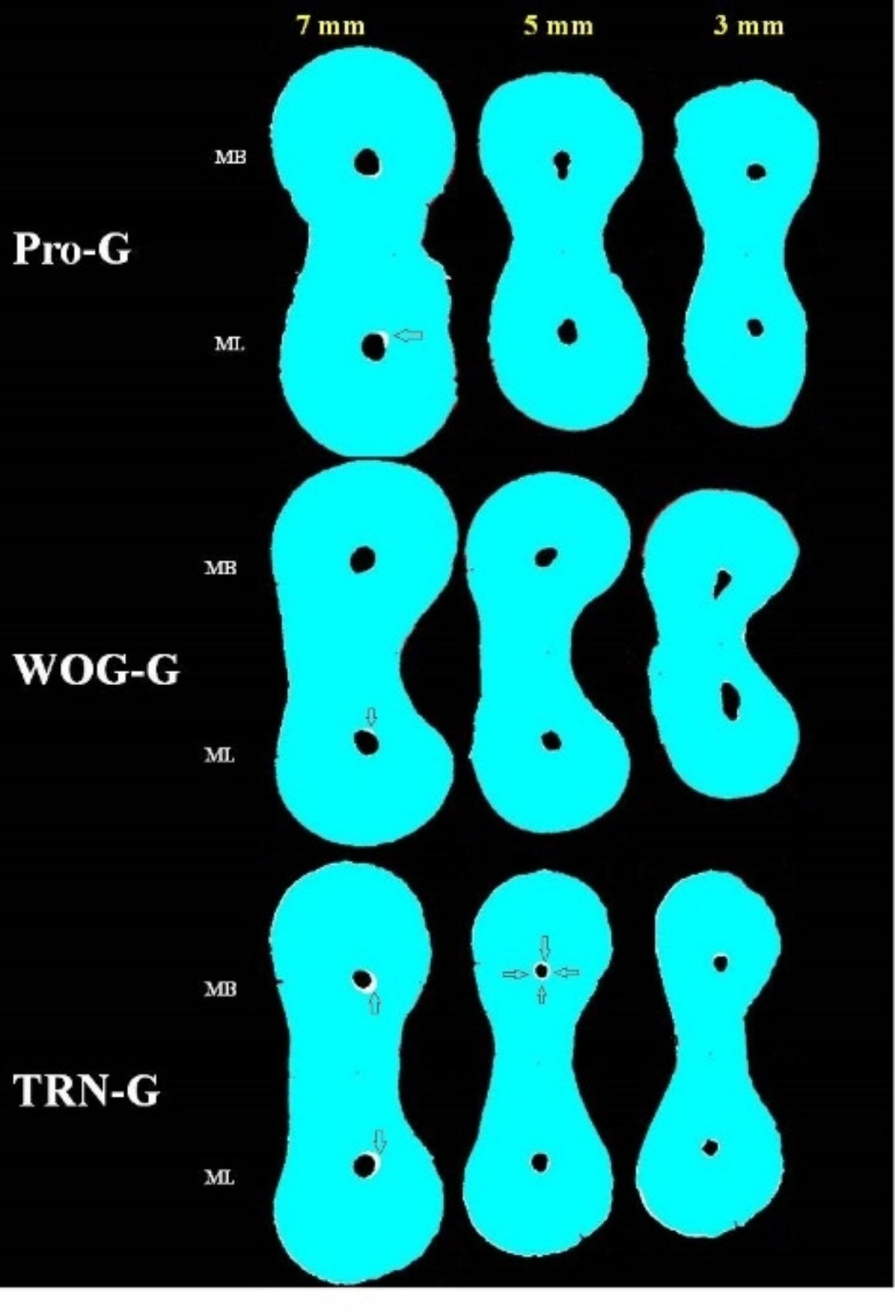
Image of 3, 5, and 7 mm sections from each group. (The arrows indicate transportation areas. In the WOG-G and Pro-G groups, buccal transportation was observed in the ML canals at the 7 mm level. In the TRN group at the 7 mm level of MB and ML canals, transportation towards the mid-mesial area was observed. Good centering ability was observed at the 5 mm level in MB canals in the TRN group.)

The first visible major apical foramen was determined to be at level 0. The three sections were determined to be 3, 5, and 7 mm above the apical foramen (Fig. 1). The transportation and centering ratio were calculated using the formulas of Ghambill et al. [20] in two mesial canals in the mesiodistal (MD) and buccolingual (BL) directions. According to this formula, as the transportation value approaches 0, transportation decreases, while ‘0’ means no transportation. The centering ability increases as the centering ratio gets closer to 1, while a ratio of ‘1’ means the best centering ability.

### Statistical analysis

Data were analysed in the R program with the WRS2 package [21, 22]. The conformity of the transportation and centering measurements to the normal distribution according to the file, canal level, and canal type parameters were evaluated with the Shapiro-Wilk test. The three-way robust ANOVA was used to compare the transportation (MD-BL) and centering (MD-BL) values that were not normally distributed according to file, canal level, and canal type (MB vs. ML). Multiple comparisons were made with the Bonferroni test. Shapiro-Wilk and ANOVA tests were used to compare the curvature and radius values pre-instrumentation. The results are presented as median (minimum-maximum). The significance level was taken as p < 0.05.

## Results

The Table 1 shows that there was no significant difference between the groups in terms of pre-instrumentation values of MB and ML canals compared to MD and BL views (p > 0.05).

The Table 3 shows the robust ANOVA Q and p values. While the file type and level parameters alone had a significant effect on MD directional transportation (p = 0.018, p = 0.006), the canal parameter did not affect MD transportation (p = 0.456). None of the parameters had an effect on BL directional transportation (p > 0.05) or on MD directional centering (p > 0.05). Whereas the file parameter had a significant effect on BL centering (p > 0.001), the level and canal parameters did not (p > 0.05).

**Table 3:** The effect of file, level and canal parameters, alone and their interactions, on transportation and canal centering ability

Transportation		Q	p
**Direction**			
**MD**	**File**	8.901	**0.018**
	**Level**	11.614	**0.006**
	**Canal** ^**MB−ML**^	0.564	0.456
	File*Level	5.862	0.253
	File* Canal ^**MB−ML**^	2.667	0.278
	Level* Canal ^**MB−ML**^	4.433	0.125
	File* Level* Canal ^**MB−ML**^	4.571	0.376
**BL**	**File**	3.307	0.210
	**Level**	2.662	0.290
	**Canal** ^**MB−ML**^	0.648	0.424
	File*Level	2.684	0.637
	File* Canal ^**MB−ML**^	0.43	0.81
	Level* Canal ^**MB−ML**^	3.67	0.174
	File* Level* Canal ^**MB−ML**^	9.106	0.073
**Centering**		**Q**	**p**
**Direction**			
**MD**	**File**	4.432	0.130
	**Level**	0.167	0.930
	**Canal** ^**MB−ML**^	0.383	0.538
	File*Level	4.967	0.328
	File* Canal ^**MB−ML**^	2.903	0.246
	Level* Canal ^**MB−ML**^	2.848	0.253
	File* Level* Canal ^**MB−ML**^	4.432	0.130
**BL**	**File**	21.597	**< 0.001**
	**Level**	3.148	0.230
	**Canal** ^**MB−ML**^	0.009	0.924
	File*Level	3.372	0.533
	File* Canal ^**MB−ML**^	0.42	0.814
	Level* Canal ^**MB−ML**^	0.518	0.777
	File* Level* Canal ^**MB−ML**^	1.768	0.794

The Table 4 shows the descriptive statistics for each group. There was no significant difference between glide path files according to level and canal type.

Regardless of canal level and canal distinction, Pro-G (0.023 mm) and WOG-G (0.021 mm) transported significantly less in the MD direction than TRN-G (0.040 mm) (p < 0.05). There was no significant difference between Pro-G and WOG-G (p > 0.05). There was no significant difference between the file types in terms of canal level and transportation in the BL direction regardless of the canal (p > 0.05) (Table 4).

Regardless of the canal and file type, significantly more MD transportation was observed at the 7 mm level than at the 3 mm level (p < 0.05). Although the amount of MD transportation was higher at the 5 mm level than the 3 mm level, the difference was not significant (p > 0.05). And although the amount of MD transportation was even higher at the 7 mm level than at the 5 mm level, again the difference was not significant (p > 0.05). There was no significant difference between levels in terms of transportation in the BL direction regardless of the canal and file type (p > 0.05) (Table 4).

Regardless of canal level and distinction, there was no significant difference between files regarding centering abilities in the MD direction (p > 0.05). In contrast, Pro-G and TRN-G showed higher centering ability in the BL direction than WOG-G (p < 0.05). There was no significant difference in this parameter between Pro-G and TRN-G (p > 0.05) (Table 4).

Regardless of the file and canal distinction, there was no significant difference between the 3, 5, and 7 mm levels in terms of centering ability in either the MD or BL directions (p > 0.05) (Table 4).

**Table 4:** Median, minimum and maximum values of the transportation and centering ability by file, canal type, and canal level (mm)

	File	Canal	Level (mm)			
			3	5	7	Total
**Transportation**						
**MD**	**Pro-G**	**MB**	0.015 (0–0.065)	0.029 (0–0.323)	0.026 (0.004–0.083)	0.025 (0–0.323)
		**ML**	0.011 (0–0.059)	0.014 (0–0.036)	0.042 (0.015–0.098)	0.018 (0–0.098)
		**Total**	0.011 (0–0.065)	0.024 (0–0.323)	0.028 (0.004–0.098)	0.023 (0–0.323)^**b**^
	**WOG-G**	**MB**	0.022 (0–0.054)	0.022 (0–0.047)	0.018 (0.007–0.051)	0.022 (0–0.054)
		**ML**	0.018 (0–0.029)	0.01 (0–0.033)	0.051 (0.003–0.149)	0.018 (0–0.149)
		**Total**	0.021 (0–0.054)	0.016 (0–0.047)	0.034 (0.003–0.149)	0.021 (0–0.149)^**b**^
	**TRN-G**	**MB**	0.032 (0.011–0.076)	0.058 (0–0.09)	0.072 (0.007–0.162)	0.044 (0–0.162)
		**ML**	0.018 (0–0.062)	0.057 (0.004–0.112)	0.054 (0–0.126)	0.029 (0–0.126)
		**Total**	0.029 (0–0.076)	0.058 (0–0.112)	0.056 (0–0.162)	0.04 (0–0.162)^**a**^
	**Total**	**MB**	0.022 (0–0.076)	0.029 (0–0.323)	0.026 (0.004–0.162)	0.026 (0–0.323)
		**ML**	0.016 (0–0.062)	0.014 (0–0.112)	0.047 (0–0.149)	0.022 (0–0.149)
		**Total**	0.019 (0–0.076)^**b**^	0.022 (0–0.323)^**ab**^	0.033 (0–0.162)^**a**^	0.025 (0–0.323)
**BL**	**Pro-G**	**MB**	0.018 (0–0.09)	0.033 (0–0.118)	0.012 (0–0.141)	0.018 (0–0.141)
		**ML**	0.025 (0–0.065)	0.021 (0–0.062)	0.047 (0–0.109)	0.025 (0–0.109)
		**Total**	0.018 (0–0.09)	0.021 (0–0.118)	0.019 (0–0.141)	0.019 (0–0.141)
	**WOG-G**	**MB**	0.033 (0.004–0.076)	0.029 (0–0.116)	0.029 (0–0.062)	0.029 (0–0.116)
		**ML**	0.022 (0–0.059)	0.026 (0.011–0.109)	0.051 (0–0.102)	0.029 (0–0.109)
		**Total**	0.027 (0–0.076)	0.027 (0–0.116)	0.033 (0–0.102)	0.029 (0–0.116)
	**TRN-G**	**MB**	0.047 (0.011–0.083)	0.021 (0–0.076)	0.051 (0–0.115)	0.029 (0–0.115)
		**ML**	0.029 (0.007–0.069)	0.025 (0.007–0.155)	0.05 (0.007–0.089)	0.029 (0.007–0.155)
		**Total**	0.029 (0.007–0.083)	0.025 (0–0.155)	0.051 (0–0.115)	0.029 (0–0.155)
	**Total**	**MB**	0.029 (0–0.09)	0.025 (0–0.118)	0.026 (0–0.141)	0.026 (0–0.141)
		**ML**	0.025 (0–0.069)	0.025 (0–0.155)	0.05 (0–0.109)	0.029 (0–0.155)
		**Total**	0.027 (0–0.09)	0.025 (0–0.155)	0.033 (0–0.141)	0.029 (0–0.155)
**Centering**						
**MD**	**Pro-G**	**MB**	0.5 (0–1)	0.301 (0–1)	0.281 (0–0.82)	0.301 (0–1)
		**ML**	0 (0–1)	0 (0–1)	0.283 (0–0.79)	0.129 (0–1)
		**Total**	0.365 (0–1)	0.287 (0–1)	0.282 (0–0.82)	0.282 (0–1)
	**WOG-G**	**MB**	0 (0–1)	0 (0–1)	0.5 (0–0.86)	0.375 (0–1)
		**ML**	0.385 (0–1)	0.667 (0–0.99)	0 (0–0.91)	0.385 (0–1)
		**Total**	0.188 (0–1)	0.53 (0–1)	0.196 (0–0.91)	0.38 (0–1)
	**TRN-G**	**MB**	0.445 (0–0.84)	0 (0–0.99)	0.242 (0–0.89)	0.286 (0–0.99)
		**ML**	0.498 (0–1)	0.273 (0–0.93)	0.464 (0.15–1)	0.394 (0–1)
		**Total**	0.456 (0–1)	0.258 (0–0.99)	0.344 (0–1)	0.354 (0–1)
	**Total**	**MB**	0.375 (0–1)	0.136 (0–1)	0.4 (0–0.89)	0.301 (0–1)
		**ML**	0.385 (0–1)	0.394 (0–1)	0.286 (0–1)	0.333 (0–1)
		**Total**	0.38 (0–1)	0.287 (0–1)	0.31 (0–1)	0.333 (0–1)
**BL**	**Pro-G**	**MB**	0 (0–1)	0.357 (0–1)	0.453 (0–1)	0.357 (0–1)
		**ML**	0 (0–1)	0.632 (0–1)	0.174 (0–1)	0 (0–1)
		**Total**	0 (0–1)	0.495 (0–1)	0.224 (0–1)	0.145 (0–1)^**a**^
	**WOG-G**	**MB**	0 (0–0.75)	0 (0–1)	0 (0–1)	0 (0–1)
		**ML**	0 (0–1)	0 (0–1)	0 (0–1)	0 (0–1)
		**Total**	0 (0–1)	0 (0–1)	0 (0–1)	0 (0–1)^**b**^
	**TRN-G**	**MB**	0 (0–0.75)	0.412 (0–1)	0.364 (0–1)	0.227 (0–1)
		**ML**	0 (0–0.64)	0 (0–0.78)	0.172 (0–0.71)	0 (0–0.78)
		**Total**	0 (0–0.75)	0.173 (0–1)	0.242 (0–1)	0.155 (0–1)^**a**^
	**Total**	**MB**	0 (0–1)	0 (0–1)	0.23 (0–1)	0 (0–1)
		**ML**	0 (0–1)	0 (0–1)	0 (0–1)	0 (0–1)
		**Total**	0 (0–1)	0 (0–1)	0.162 (0–1)	0 (0–1)

a-b: There is no significant difference between groups with the same letter (p > 0.05).

## Discussion

Increasing the reliability and effectiveness of larger instruments during root canal preparation is a hot topic in the current endodontic shaping protocol. The development of NiTi instrumentation systems supports this concept with different glide path instruments. Therefore, this present study compared the shaping ability of WOG-G, TRN-G, and Pro-G with micro-CT imaging in mandibular mesial canals.

According to the results obtained in the present study, Pro-G and WOG-G transported significantly less in the MD direction than TRN-G. However, there was no significant difference between files regarding centering abilities in the MD direction. Despite no significant difference between groups in terms of transportation in the BL direction, Pro-G and TRN-G showed higher centering ability in the BL direction than WOG-G. Furthermore, no significant difference was found among the experimental groups regarding centering ability at any level. In addition, more MD transportation was observed in the coronal third than in the apical third. Therefore, the null hypothesis of this study was rejected. Overall, transportation was observed to be lower than 0.1 mm in all groups, which the high flexibility of the tested instruments can explain. Also, glide path instruments have an active role in preliminary shaping root canals; they have been designed with slim size and low taper. TRN-G provided higher transportation than other tested systems; tip size differences may explain this difference. TRN-G has a 0.17 mm tip diameter and a bigger tip size than others. According to the authors of this study, the values are so small that they can be considered irrelevant from a clinical perspective.

To the author’s knowledge, no previous studies have evaluated the shaping ability of TRN-G instruments, so we were unable to compare the current study’s results directly with another study. Besides, only two studies have assessed the transportation and centering ability of Pro-G and WOG-G [14, 16]. Miró et al. reported no significant differences in canal transportation, but WOG-G had better centering ability in the apical third than Pro-G [14]. Aydın et al. [16] showed that WOG-G had a better centering ability and caused less transportation in the middle and coronal thirds than Pro-G. In our study, the Pro-G instrument caused small canal transportation that was more directed in the MD direction and had better centering ability in the BL direction. Consistent with our results, the Pro-G instrument’s canal transportation and centering ability have been evaluated in a few studies reporting small transportation [11, 16]. It should be noted that Miró et al. evaluated these parameters in the mesiobuccal root canals of maxillary molars [14]. Although the methodologies of the studies are similar (same instruments, distances from the apical, using micro-CT), operator skill may have been the cause of differences in the results. As mentioned in a literature review, omissions in the irrigation protocol and the operator’s experience determining the most appropriate preparation technique for each situation should be considered variables in shaping ability studies [23].

In the present study, despite the differences in the kinematics, root canals prepared with glide path instruments showed similar centering ability in all groups at all measuring points. This may be attributed to the fact that heat-treated NiTi glide path instruments tend to maintain their original shape during instrumentation of the mandibular mesial root canals [24, 25], and because all glide path instruments used in this study were made of heat-treated NiTi alloy available in the martensitic phase, which increases instrument flexibility [26]. WOG-G instruments (which undergo the Gold treatment), Pro-G instruments (which undergo the M-wire treatment), and the TRN-G instruments (which undergo a new thermal treatment ) were evaluated [19]. Despite not being fully known, Dias et al. stated that heat-treated NiTi alloy of TRN systems follows the current trend towards a predominance of martensite phase [19].

The cross-sectional design and stiffness of the instruments have been identified as factors potentially influencing preparation outcomes [27, 28]. Three different files (Pro-G, WOG-G, and TRN-G) for creating a glide path were examined in this study. Despite their small size, the tested instruments have different cross-sectional designs and tapers: a parallelogram with progressively tapering (of WOG-G), a square with progressively tapering (of Pro-G), and an off-centered parallelogram with variable tapering (TRN-G). These features effectively increase flexibility and adaptation to the curved canal anatomy [12, 29]. TRN-G, Pro-G, and WOG-G have similar nominal sizes and tapers. Since these various features cannot be fully isolated, this could be considered a limitation in the unambiguous interpretation of the results [30].

Researchers use mandibular mesial root canals to mimic clinical conditions, thanks to the teeth’s anatomy, the dentin’s hardness, and the concave and convex irregularities on the canal surface [31]. For this reason, mandibular mesial roots with two separate canals with moderate curvature were preferred during sample selection, and this is one of the essential stages of the methodology, as the degree of curvature, length, and location of the root canal will influence the shaping ability [32]. Also, it is essential to note that only the transportation and centering ability of tested glide path instruments were assessed. In the present study, sample selection was done on the CBCT images of teeth using previously established methods [33, 34]. So, one of the limitations of this in-vitro study could not be checked canal shape in the sample selection stage.

Micro-CT provides the best and most precise assessment of the biomechanical preparation of the root canal system for evaluating the shaping ability of different NiTi systems, non-destructively and with a high resolution [23, 35, 36]. Thus, the present study used micro-CT scanning to assess canal transportation and centering ability over the apical, middle, and coronal root canal levels (3, 5, and 7 mm from the root apex). In addition, these measurements represent where curvatures with high vulnerability to iatrogenic mishaps typically exist [37].

## Conclusion

Glide path preparation with NiTi systems is recommended for efficient and safe root canal preparation. This study confirms that the tested glide path files are reliable in terms of transportation and centering.

## Data Availability

The datasets analysed during the current study are available from the corresponding author on reasonable request.

## List of abbreviations

TRN-G: TruNatomy Glider
WOG-G: WaveOne Gold Glider
Pro-G: ProGlider
Micro-CT: micro-computed tomography
MB: mesiobuccal
ML: mesiolingual
MD: mesiodistal
BL: buccolingual
NiTi: nickel-titanium
WL: working length
NaOCl: sodium hypochlorite
